# 3D ultrasound strain imaging of puborectal muscle with and without unilateral avulsion

**DOI:** 10.1007/s00192-023-05498-1

**Published:** 2023-04-14

**Authors:** Shreya Das, Gijs A. G. M. Hendriks, Frieda van den Noort, Claudia Manzini, C. H. van der Vaart, Chris L. de Korte

**Affiliations:** 1 Medical Ultrasound Imaging Center, Department of Medical Imaging, Radboud University Medical Center, Geert Grooteplein Zuid 10 (767), PO Box 9101 (766), 6500 HB Nijmegen, The Netherlands; 2 Robotics and Mechatronics, Technical Medical Center, University of Twente, Enschede, The Netherlands; 3 Department of Reproductive Medicine and Gynecology, University Medical Center, Utrecht, The Netherlands; 4 Physics of Fluids, TechMed Center, University of Twente, Enschede, The Netherlands

**Keywords:** Ultrasound, Puborectal muscle, Unilateral avulsion

## Abstract

**Introduction and hypothesis:**

The puborectal muscle (PRM), one of the female pelvic floor (PF) muscles, can get damaged during vaginal delivery, leading to disorders such as pelvic organ prolapse. Current diagnosis involves ultrasound (US) imaging of the female PF muscles, but functional information is limited. Previously, we developed a method for strain imaging of the PRM from US images in order to obtain functional information. In this article, we hypothesize that strain in the PRM would differ from intact to the avulsed end.

**Methods:**

We calculated strain in PRMs at maximum contraction, along their muscle fiber direction, from US images of two groups of women, which consisted of women with intact (*n*_1_ = 8) and avulsed PRMs (unilateral) (*n*_2_ = 10). Normalized strain ratios between both ends of the PRM (avulsed or intact) and the mid region were calculated. Subsequently, the difference in ratio between the avulsed and intact PRMs was determined.

**Results:**

We observe from the obtained results that the contraction/strain pattern of intact and undamaged PRMs is different from PRMs with unilateral avulsion. Normalized strain ratios between avulsed and intact PRMs were statistically significant (*p* = 0.04).

**Conclusion:**

In this pilot study, we were able to show that US strain imaging of PRMs can show differences between intact PRMs and PRMs with unilateral avulsion.

## Introduction

The female pelvic floor (PF) muscles, collectively known as the levator ani muscles (LAM), provide support to the overlying PF organs and also allow the passage of the baby during vaginal delivery. The puborectal muscle (PRM) together with pubococcygeus and iliococcygeus muscles forms the LAM. Computer simulation of vaginal birth shows that PRM has to stretch up to 2.28 times [1]. Due to this elongation, the entire LAM including the PRM can get damaged, leading to pelvic floor dysfunction [2–5, 25]. The prevalence of pelvic floor dysfunction increases with age [6]. The extent of induced damage determines the inability of the LAM to provide support to the PF organs, which can lead to pelvic organ prolapse (POP) [14, 15].

Information about the extent and the location of PRM damage would determine whether the first line of treatment, i.e., physiotherapy, would be effective. It would also determine the necessity of training specific muscles such as PRM, depending on whether it is functionally damaged.

The current methods of detecting LAM damage involve imaging of it by ultrasound (US) or magnetic resonance imaging (MRI) [7–11]. Dynamic imaging of PF muscles enables characteristic measurements of certain anatomical structures. For example, by dynamic imaging of a healthy contraction, substantial shortening of the levator hiatus in the sagittal plane and a change in the angle between levator plane and pubic symphysis (PS) can be quantified. Other PF organs, including the uterus, bladder, and urethra, will be displaced cranially, as well as compression of the urethra, vagina, and anorectal junction [12]. The deviations from these characteristic measurements of anatomical structures such as the anorectal angle, perimeter of the PRM at contraction, and hiatal area at Valsalva can be measured through imaging [12, 13]. Diagnosis of PRM damage is dependent on these measurements together with the symptoms experienced by the woman. Imaging, whether through US or MRI, is most effective when the damage in the muscle or the group of muscles is extensive and has been termed as macroscopically visible tears [14]. Unilateral or bilateral avulsions are consequences of macroscopically visible tears in the PRM.

### US imaging of PF muscles and its limitations

Transperineal US (TPUS) is frequently used for female PF imaging because of its versatility and real-time capabilities [7]. To obtain information about the state of the PF muscles, US acquisition from these muscles is from rest to contraction. Although information is obtained over the contraction cycle, only global geometrical changes can be determined from the static 2D images forming the 3D volumes. Thus only indirect information on the contractile properties can be deduced. Functional information cannot be deduced [14, 15].

### Strain imaging of PRM using 3D US

A certain analogy can be derived from cardiac strain imaging or echocardiography, to explain why deformation and strain imaging might be a good alternative to assess functional properties. Myocardial deformation is a result of contraction and relaxation of the cardiac muscle fibers in the longitudinal, circumferential, and radial directions during a cardiac cycle, along with a twisting motion. Therefore, the measurements of these deformations are typically made along these three axes, resulting in longitudinal, circumferential, and radial strain respectively. Strain is advantageous because it can assess regional as well as global function and is less susceptible to cardiac translation, imaging angle, and measurement error than conventional systolic function measurements. 3D speckle tracking echocardiography allows for the simultaneous measurement of global and regional longitudinal, circumferential, and radial strains. Among these, global longitudinal strain is emerging as an important parameter of prognostic value in patients with (risk of) cardiovascular disease [16, 17].

Analogous to the longitudinal cardiac strain, in this work we have estimated strain along the muscle fiber direction of the PRM, using 3D speckle tracking of US images [18]. PRM imaged by TPUS involves data acquisition from rest to maximum voluntary contraction and then back to rest. Quantifying the deformation of the muscle provides functional information of the PRM.

### Aim of the study

The aim of the study is to investigate if contraction/strain patterns of intact PRMs and PRMs with unilateral avulsion show differences. Our hypothesis is that intact or undamaged PRMs will have different contraction patterns than PRMs with unilateral avulsion in which scar or connective tissue formation can be present.

## Methods

### Data acquisition

Dynamic 3D TPUS volumes were acquired using a Philips X6-1 matrix transducer connected to an EPIQ 7G US machine (Philips Healthcare, Bothell, WA, USA) at Bergman Clinics, Hilversum, The Netherlands. Total acquisition length was 11 to 15 s at a volume rate of 1.5 Hz. The data were stored in the digital imaging and communications in medicine (DICOM) format. US data were obtained over time from women (*n*_1_ = 8) who had intact PRMs (although with overactive pelvic floor) and from women (*n*_2_ = 10) who had unilateral avulsion. Presence of unilateral avulsion was diagnosed by the clinician. Complete unilateral avulsion was defined as levator–urethra gap of ≥ 25 mm from the urethra on the left or right side, as measured from three central 2D US slices at the minimal hiatal area, during maximum contraction [14]. In all women, US volumes were recorded from rest to maximum contraction, as the women were asked to actively contract their PF muscles. To ensure that the women could contract their PF muscles, the clinician acquired the data while the US images were being observed on the screen of the US machine, which was used as a bio-feedback. The Medical Research Ethics Committee of UMC Utrecht exempted the project from approval, and all women signed appropriate research consent forms.

Women, with differences in age, BMI, and parity have been included in this pilot study to investigate whether the developed method produces different values for getting strain values for intact and undamaged PRMs and damaged PRMs.

### Post-processing of US volumes

**Fig. 1:**
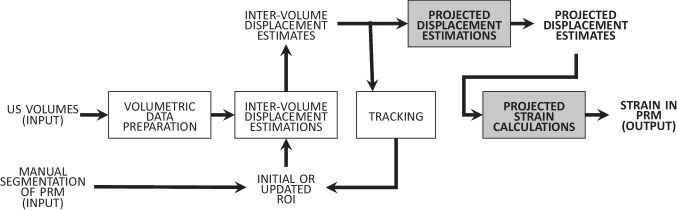
Block diagram 1. The blocks in *white* have been explained in our previous publication [19]. The blocks in *grey with bold letters* have been explained in the current section

The next step after data acquisition is to calculate strain in the PRMs using the obtained US volumes (DICOM). The method to calculate strain is explained thoroughly in our previous publication and is summarized in Fig. 1 [19]. In short, inter-volume displacements were calculated between subsequent recorded volumes and used to update the segmentations of the PRMs. Those intervolume displacements were accumulated to calculate strain during muscle contraction.

Since the objective of this work is to detect whether there might be differences in the contraction pattern of the PRM, the strain in the muscle fiber direction is determined. However, since the muscle fiber direction is dependent on the location, the displacement estimates were determined in the x, y, and z directions. From that, strain along the muscle fiber direction have been obtained.

**Fig. 2:**
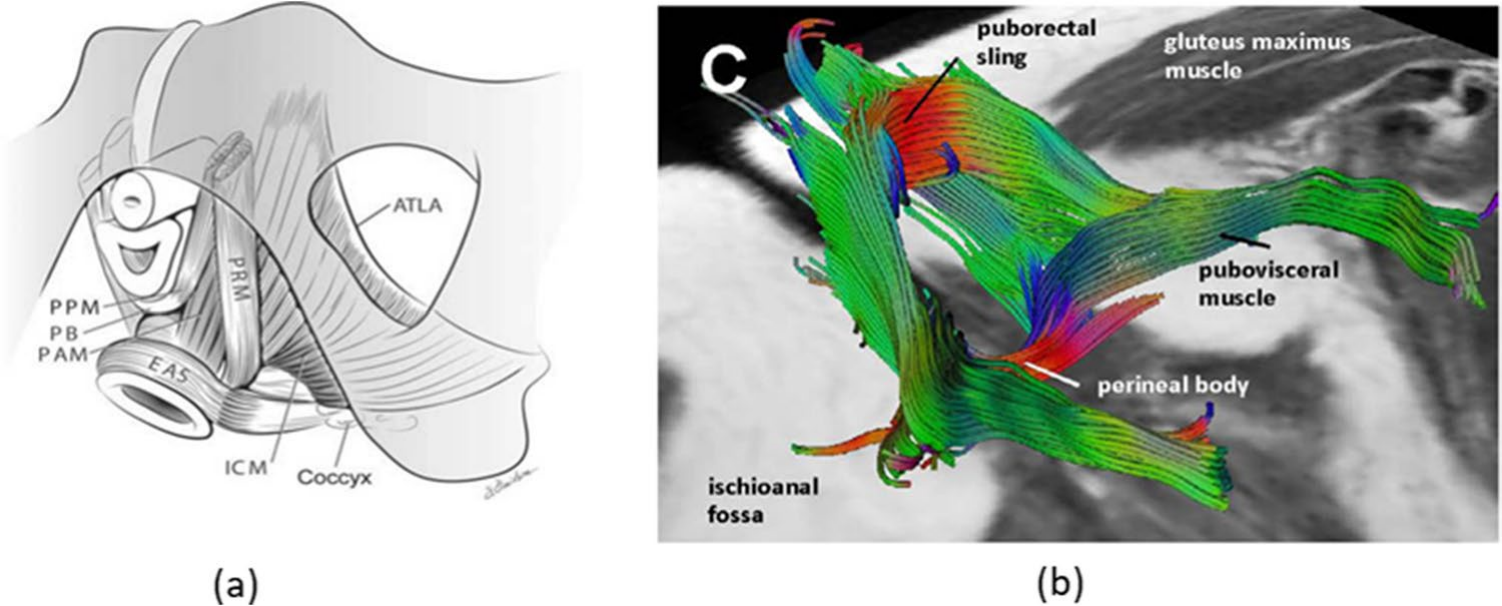
**a** Pelvic floor anatomy showing PRM [20]. Permission granted by the publisher to use this image. **b** MR tractography showing the muscle fiber orientation of the PRM and the puborectal sling [21]. Permission granted by the publisher to use this image

Information about the muscle fiber direction is not provided by studies on gross anatomy [20]. However, this information has been determined by MR fiber tractography [21]. According to MR tractography of female PF, the fiber directions are such that the individual fibers are connected to the two parts of the bone pubic symphysis (PS), and they curve around to form the sling of the muscle as shown in Fig. 2a and b.

Therefore, we modified the original method to assess strain along the fiber direction (gray blocks of the block diagram in Fig. 1). As shown in this block diagram, the ‘inter-volume displacement estimates’ at a certain time point or volume number (variable from woman to woman) were used to calculate the ‘projected displacements’ along the fiber direction. Therefore, the center line of the PRM was calculated, which acted as reference for the muscle fiber direction.

**Fig. 3:**
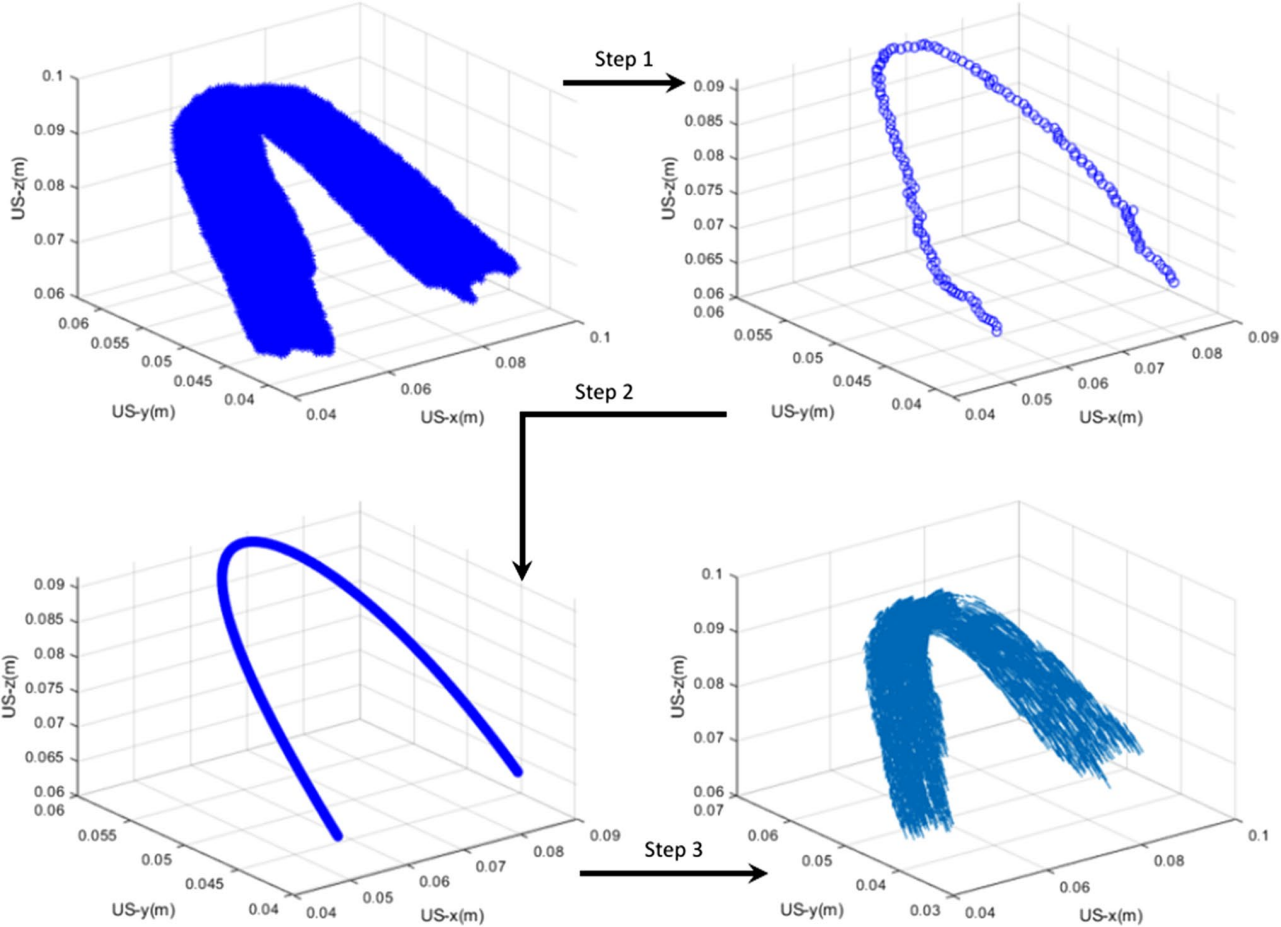
An example of a segmented PRM (in 3D), showing the center line through it and center line after curve fitting and scaling (Steps 1–3). The last figure shows the displacement estimates projected on the center line

The center line of the muscle was obtained in three steps. In the first step, the ‘manual segmentation of PRM’, or ‘updated ROI’ as shown in Fig. 3, was eroded over its entire length. This allowed us to obtain a connected curve or center line over the length of PRM. In the second step, this connected curve was fitted to a polynomial curve to smooth the obtained curve and to remove outliers. In the third step, the accumulated displacements (x, y, and z directions) were projected to the closest point on this central axis using vector projections [22].

Finally, strain was calculated by the projected displacements using a 2 × 2 x 2 least-squared strain estimator (LSQSE) [23]. These calculations were performed while correcting for the change in angle or direction of the previously calculated center line of the PRM. Thus, we obtained the projected strain values for the PRM at a certain volume. These projected displacements were calculated for all volumes, from rest to contraction.

### Normalized ratio of strain percentage in right vs left ends of PRM

Anatomically, the muscle fibers in the PRM are approximately parallel to the US transducer direction in the two connected ends, when imaged in TPUS. The muscle fibers in the mid region or’sling’ of the PRM are approximately perpendicular to the US transducer direction. Since we are interested in the effect of avulsion on the local contraction properties of the PRM, we have divided the whole PRM into three parts: the two ends, that are in undamaged condition connected to the PS, and the mid region between these two ends, containing the ‘sling’. The reason for choosing three regions in the PRM is based on the observation (later shown in the Results section, Fig. 5a) that in the case of intact PRMs, the two regions near the bone show similar strain and the mid-region show different strain. The division of the muscle into three regions enable us to relate the strain in the ends of the PRM with that of the mid-region through normalized strain ratio.

**Fig. 4:**
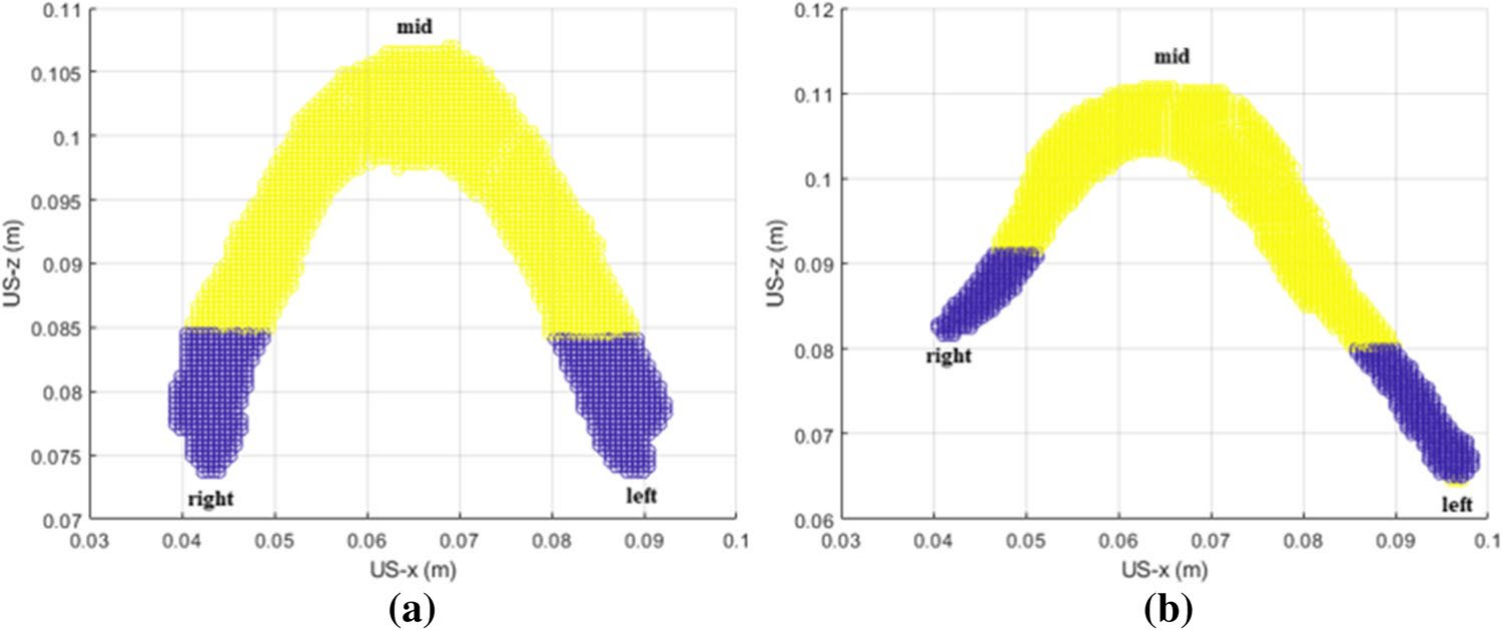
Three regions (shown in XZ plane, axial view) within the PRM with the right, left (*blue*) and mid regions (*yellow*) labeled. **a** Example showing intact PRM. **b** Example showing PRM with unilateral avulsion

Since the PRM for each woman has slightly different size and volume; each PRM has been individually divided into three subdivisions. The combined volume of both the subdivisions of the PRMs, which includes the connection to the PS (shown in blue in Fig. 4a and b), was approximately one-third of the total volume of the PRM. These three subdivisions have been done for both intact PRMs and PRMs with unilateral avulsion. Examples of these sub-divisions or regions are shown in Fig. 4a and b.

For the analysis, as mentioned earlier, strain values in the three regions were used. As a first step, medians of the strain values were obtained for the three regions for all the women. Since the contraction of the PRM was widely variable among women, a wide range of strain values was obtained. To correct for this effect, the difference in strain values for the two ends was normalized by relating it to average difference in strain between the mid region and the average of the two ends according to Eq. 1.

1 $$Normalized\;strain\;ratio=abs\left(\varepsilon_{right}-\varepsilon_{left}\right)/abs\left(\varepsilon_{mid}-\left(\varepsilon_{right}+\varepsilon_{left}\right)/2\right)$$

where,

Ɛ_right_: - median of the percentage of strain in right region of PRM,
Ɛ_left_: - median of the percentage of strain in left region of PRM,
Ɛ_mid_: - median of the percentage of strain in mid region of PRM.

The abbreviation’abs’ denotes absolute values.

### Statistical analysis

The normalized strain ratio was calculated for all women with and without unilateral avulsion (two independent and non-paired groups). Since the normalized strain ratios were not normally distributed, non-parametric statistical analysis had been performed. The Shapiro–Wilk test of normality was used to verify non-normality. Statistical significance has been calculated using the Mann–Whitney U-test, and a *p*-value  < 0.05 was considered significant [24].

*P*-values and medians for the two groups for age, BMI, and parity have been calculated and shown in Table 1. Since ages and BMIs for the two groups followed normal distribution, *t*-tests were used for calculating *p*-values. To calculate the *p*-value for parity, the Mann–Whitney U-test was used, since parity values for the two groups did not follow normal distribution.

## Results

The developed method had been applied on all the datasets acquired from women with intact and undamaged PRMs (*n*_1_ = 8) and women with unilateral avulsion (*n*_2_ = 10). The demographic characteristics of all these women is shown in Table 1. The median values of age, BMI, parity, and additional information about delivery for all the women included are listed as indicators of the health of their PF muscles. Women with intact PRMs are in general younger and have less parity, as shown in the medians. The median of BMI between the two groups shows lower median for the group with intact PRMs, although were not significantly different. In this work, the effects of different age, parity, and BMI have not been analyzed; they can only be done on a large-scale study. Thus, statistical significance for these parameters between the two groups have also been not considered.

**Table 1:** Demographics characteristics of all inclusions and respective *p*-values

	Women with intact PRMs (*n*_1_ = 8)	Women with unilateral avulsion of PRMs (*n*_2_ = 10)	*P*-value
Median of age	34	58	0.01
Median of BMI	20.9	24.4	0.07
Parity	0–1	1–3	< 0.01
Additional Information	Seven women nulliparous, one woman vaginally nulliparous with primary C-section	One woman with one vaginal delivery (primary C-section), one woman with one vaginal delivery (vacuum extraction), six women with two vaginal deliveries, two women with three vaginal deliveries	-

**Fig. 5:**
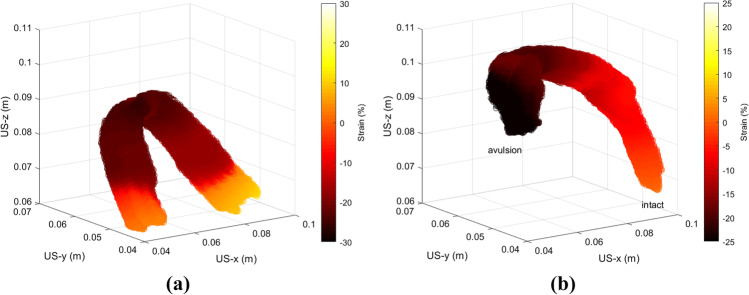
Percentage of strain at maximum contraction (shown in 3D) in two women with **a** intact PRM and **b** unilateral avulsion of PRM. The avulsed and intact ends of the avulsed PRM are labeled. Axes x, y and z are in meters, and the *color bars* show percentage of strain from negative (*dark red*) to positive (*yellow*)

Figure 5a and b show typical projected strains of an intact PRM and a PRM with unilateral avulsion respectively. The projection of the strain on the central axis resembles the strain in and along the muscle fiber direction. The intact PRM shows that high negative strain values (indicating high levels of contraction) are present in the mid region, and the strain values at both ends show positive strain values. In the case of PRM with unilateral avulsion, it can be observed that the strain pattern differs from the intact and undamaged PRM. Here, strains in the avulsed end and the mid region are negative. In the intact end of the PRM, strain is positive, similar to the two intact ends in case of an intact PRM. We observed similar findings for the strain values in all women in the two groups, as observed in these two examples.

**Fig. 6:**
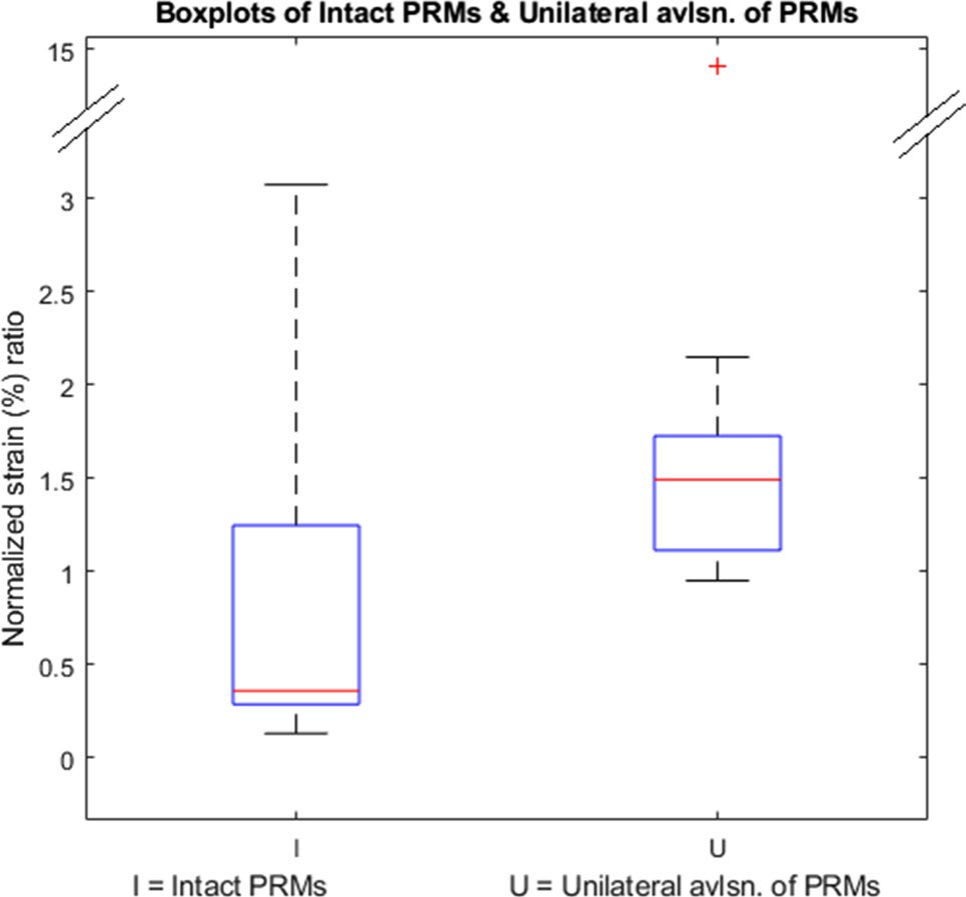
Boxplots showing normalized strain ratio for women with intact PRMs and women with unilateral avulsion

In Fig. 6, two boxplots are shown of the normalized strain ratio obtained using Eq. 1 in the two groups of women. In case of women who do not have an avulsion, the ratios are in general less than 1, which means that the two ends of the PRMs show similar strain values. In case of women with unilateral avulsion, the ratio in general is larger than 1. Values more than 1 means that the difference between the two ends is greater than the difference between the strain of the mid region and the strain in the two ends. In other words, it means that these particular PRMs have a large difference in strain values between the two ends.

In the case of women with intact or undamaged PRMs, the median of the normalized strain percentage ratio is 0.36, with the upper and lower limits being 3.07 and 0.13 respectively. In the case of women with unilateral avulsion the median is 1.54, with upper and lower limits being 14.59 and 0.95 respectively. The difference in the medians between these two groups of women is represented in the boxplots.

The normalized strain ratio is within 1, for six out of the eight women with undamaged PRMs. For two women in this group, we found that the normalized strain ratio is more than one (1.96 and 3.07).

In the group of women with unilateral avulsion, the normalized strain ratio is larger than 1 for nine out of ten women. For one of the women, the ratio is 0.95.

Non-parametric statistical analysis using the Mann–Whitney U-test shows a significant difference (*p* = 0.04), demonstrating that women with unilateral avulsion have a higher ratio than women with intact PRMs.

## Discussion

The principal finding of this pilot study is that the strain pattern at contraction in the avulsed end of the PRM is different from the intact end, whereas strain patterns were similar in both ends of intact and undamaged PRMs.

PRM is part of LAM. The function of the LAM is to maintain equilibrium by balancing the hydrostatic pressure of the overlying pelvic organs and also to adjust to variations in posture [1, 2, 25]. LAM consists of type 1 striated muscle fibers. Striated muscles are most prone to injury when they are forcibly lengthened, such as in vaginal birth [1, 2]. Earlier studies have demonstrated that LAM defects occur after vaginal birth which can confer a four- to eleven-fold increase in risk for developing prolapse among parous women [2]. LAM damage is avulsion of the PRM [14]. This damage can be imaged by functional imaging.

Current literature already explains the term functional imaging using MRI [26]. Functional US imaging of the female PF can be defined as US imaging of the PF while the woman voluntarily contracts or undergoes a Valsalva maneuver (VM) of their PF muscles. In recent functional studies of the PF, shear wave elastography (SWE) has been studied, and it has been reported that it is possible to measure the shear modulus of the LAM, at rest and at VM [27]. Another method reported in literature is representative finite element model (FEM) of the PF based on anatomy from a 2D or 3D level [28, 29]. These studies have used MRI imaging of PF at VM instead of contraction of the LAM. Since, we used 3D strain as measure for contraction, we could not directly compare our results with already published results.

In the obtained results, in case of the group of women with intact and undamaged PRMs, it can be observed in Fig. 5a that the contraction started at the mid region or slings of the PRMs. The negative strain in the sling shows contraction of this part of the PRM. The two ends which are connected to the PS show positive strain, which indicates elongation of these two parts. This indicates that the contraction of the PRM is dominated by the central area, whereas both connected ends appear to elongate, as indicated by the positive strain values.

Our results, as shown in Fig. 5b, showed that in case of strain in PRMs in women with unilateral avulsion, negative strain was obtained in the mid-regions as well as the avulsed ends of the PRMs. The possible explanation for this is that the avulsed end could contract along with the mid region to which it was connected, but not elongate since it was not connected to the bone. The intact ends of these PRMs still showed positive strain indicating elongation. This revealed a different contraction pattern when compared with strain images of PRMs with no damage, where both the intact ends of the muscle showed positive strain or elongation. Thus, we obtained different contraction patterns in the two groups of women.

The normalized strain ratio is within 1, for six out of the eight women with undamaged PRMs. This indicates that the strain in the two connected ends of the PRM is similar. This is also expected, because the ends of the muscle are intact and undamaged, resulting in similar strain. For two women in this group, we found that the normalized strain ratio is more than one (1.96 and 3.07). Since, our study is a pilot study, the cause for this higher ratio could not be determined.

In the group of women with unilateral avulsion, the normalized strain ratio is larger than 1 for nine out of ten women. This indicates that the strain in the avulsed end of the muscle is more negative than strain in the intact end. For one of these women, the ratio is marginally less than 1.

Although we had to perform a non-parametric statistical test due to small sample sizes, we were still able to show that the strain ratio is significantly (*p* = 0.04) different between avulsed and intact PRM’s.

### Clinical significance

Potential clinical significance of our work can be stated as two-fold. Firstly, recognizing which part of the muscle is dysfunctional may be of benefit for physiotherapists treating women with pelvic floor disorders. Also, recognizing that there is presence of severe structural damage may well change the policy of having pelvic floor muscle training in every women with pelvic floor disorders.

Secondly, when clinicians look at regenerative action for the muscle after delivery, it would be possible to determine if there has been functional improvement of the muscle after training. Changes in strain in the muscle as observed from before to after training could make it possible to tailor the training to obtain optimal benefit.

### Limitations and future work

There are three limitations of the work. Firstly, the tracking of the PRM that was an integral part of the method was only visually verified on B-mode US volumes. However, since the technology is based on similar principles as applied for extensively validated cardiac strain imaging techniques, and the contraction patterns are in accordance with physiologic assumptions, it can be concluded that the performed analysis is valid. Secondly, currently our method is based on in-house-developed software for displacement estimation, which makes the method computationally extensive. However, since 3D cardiac strain estimation is almost feasible online, we expect our analysis can also be made nearly real-time. Thirdly, the segmentation of the PRMs at rest was performed manually, which could be extended to automated as is demonstrated in literature [30].

To further investigate PF muscles through 3D strain, future studies should include larger sample sizes and an effort to reduce the computation time for calculation of the strain values. Also, other muscles of the LAM apart from PRM should be studied to learn how the surrounding muscles behave in relation to each other, in both groups of women.

## Conclusion

We were able to show in this pilot study that women with unilateral avulsion of their PRMs show different strain pattern than women with intact PRMs.
